# Effects of soluble epoxide hydrolase inhibitor on the expression of fatty acid synthase in peripheral blood mononuclear cell in patients with acute coronary syndrome

**DOI:** 10.1186/1476-511X-12-3

**Published:** 2013-01-10

**Authors:** Xuan Zhao, Jian-qing Du, Dan-yan Xu, Shui-ping Zhao

**Affiliations:** 1 Department of Cardiology & Internal Medicine, Second Xiangya Hospital, Central South University, 139 Middle Ren min Road, Changsha, 410011, PR China

**Keywords:** Soluble epoxide hydrolase inhibitor, Fatty acid synthase, Acute coronary syndrome

## Abstract

**Background:**

Researches have shown that soluble epoxide hydrolase inhibitors (sEHi) can protect against the development of atherosclerosis. Simultaneously, emerging evidences have implicated the association between fatty acid synthase (FAS) and acute coronary syndrome (ACS). We tested the hypothesis that sEHi could reduce the occurrence of ACS by regulating FAS.

**Methods:**

Hospitalized ACS patients were selected as the ACS group (n = 65) while healthy normal subjects as the control group (n = 65). The blood levels of lipoproteins, fasting glucose, myocardial enzyme and high-sensitivity C-reactive protein (hs-CRP) were measured within 24 hours after admission. The peripheral blood mononuclear cells (PBMCs) were isolated and cultured. *Trans*-4-[4-(3-Adamantan-1-ylureido)cyclohexyloxy] benzoic acid (*t*-AUCB), a kind of sEHi, was then added to cells in various concentrations (0, 10, 50, 100 μmol/L). The expression of FAS, interleukin-6 (IL-6) mRNA and protein was detected by real-time PCR or Western blot, respectively.

**Results:**

(1) Compared with the control group, the serum concentration of hs-CRP in the ACS group was increased (*P*<0.05). The expression of FAS, IL-6 mRNA and protein were significantly increased in PBMCs from the ACS group (all *P*<0.05). Moreover, the levels of FAS and IL-6 mRNA were positively correlated with the serum concentration of hs-CRP (r = 0.685, *P*<0.01; r = 0.715, *P*<0.01) respectively. (2) The expression of FAS, IL-6 mRNA and protein in PBMCs from the ACS group were dose-dependently inhibited by sEHi (all *P*<0.05).

**Conclusions:**

sEH inhibition regulated FAS and inhibited inflammation in cultured PBMCs from ACS patients, a mechanism that might prevent rupture of atherosclerotic lesions and protect against development of ACS.

## Background

Acute coronary syndrome (ACS) is the leading cause of death and loss of life worldwide. ACS usually occurs when plaques are suddenly ruptured [1-4]. Numerous studies have shown that the concentrations of saturated fatty acid (SFA) in plaques and the thickness of the fibrous cap are associated with the formation of disrupted plaques [5]. Greater concentrations of SFA (3.5 versus 2.9, *P* < 0.05) have been found at the edges of disrupted plaques than the center, which could reflect that increased SFA levels adversely influenced plaque stability [6,7]. Moreover, Felton et al. [7] stated that increased SFA levels at the edges of advanced plaques was inversely associated with cap thickness, and therefore might reflect a predisposition to plaque rupture. The synthesis of SFA is an energy-consuming process that requires the multifunctional enzyme, fatty-acid synthase (FAS) [8]. It has been suggested that FAS plays an important role in the development of ACS by regulating the synthesis of SFA.

Evidence suggests that FAS is the key enzyme that regulates differentiation of the monocyte into the macrophage, and the inhibition of FAS limits phagocytosis by macrophages [9]. Indeed, macrophages have been shown to ingest oxidized low-density lipoprotein cholesterol (ox-LDL-C) through phagocytosis in the subendocardium, which is the basis of the development of atherosclerosis. Moreover, macrophages release lytic enzymes that degrade the fibrous cap, resulting in plaque instability and rupture [10]. Therefore, the inhibition of FAS could decrease ACS by reducing the number of macrophages present in the plaque and preventing phagocytosis by macrophages.

Furthermore, inflammation also plays a key role in development of ACS [11,12]. Consequently, it is not surprising that biomarkers of inflammation, such as high-sensitive C-reactive protein (hs-CRP) and interleukin-6 (IL-6), have been used to indicate inflammatory status in these diseases. Likewise, the concentration of FAS was positively correlated with the levels of inflammatory factors *in vivo*[13,14]. One study showed that inflammation upregulated FAS expression at the levels of both mRNA and protein, and stimulated lipogenesis in non-adipose tissues, which caused ectopic lipid deposition [15]. Taken together, these data suggest that FAS is associated with plaque rupture mediated by regulating lipid metabolism and inflammatory processes.

Soluble epoxide hydrolase (sEH) is an emerging target for pharmacological treatment of cardiovascular diseases because the inhibition of sEH leads to increased circulating levels of epoxyeicosatrienoic acids (EETs) and other fatty acid epoxides, which mediate endothelium-dependent vasodilation, promote angiogenesis and have anti-inflammatory properties [16-19]. sEH inhibitors (sEHi) were originally developed as antihypertensive and anti-inflammatory agents [20-23]. Moreover, Ulu et al. [24] found that sEHi could reduce atherosclerotic plaque formation in the apoE-KO mouse model. However, no studies exist regarding sEHi for the treatment of ACS.

Based on the evidence given above, we expected that sEHi might affect the development of ACS via stabilization of the plaque and anti-inflammation by regulating FAS. In our study, we showed that (1) The expression levels of FAS, IL-6 mRNA and protein in the ACS group were obviously increased. (2) The expression of FAS mRNA and protein in peripheral blood mononuclear cells (PBMCs) from the ACS group were dose-dependently inhibited by sEHi. Therefore, it was implied that sEHi might have effects on prevention of ACS.

## Methods and materials

### Subjects

According to diagnosis and classifications standard of ACS by the WHO [25], 65 patients with acute coronary syndrome from 49.8 to 75.2 years old (64.8 ± 12.1 years), including 38 men and 27 women, were admitted to the ACS group. The control group comprised 65 healthy volunteers from 53.4 to 73.2 years old (65.2 ± 10.2 years), including 39 men and 26 women. Subjects were excluded from the study if they had severe liver and kidney diseases, lung diseases, fracture, carcinoma, any kind of infectious disease, autoimmunity disease or combined acute complications. All patients provided written informed consent and the study was approved by the Ethics Committee of Second Xiangya Hospital, Central South University, Changsha, Hunan Province, China.

### Methods

#### Biochemical analysis

All the subjects selected were admitted to hospital within 24 hours, and blood was taken for isolation and culturing of PBMCs. Routine blood, serum concentrations of glucose, hs-CRP, total cholesterol (TC), triglycerides (TG), LDL-C and high density lipoprotein cholesterol (HDL-C) were measured the following morning after fasting for 10 hours. The methods for measurement of biochemical variables, including fasting glucose concentrations, fasting lipoprotein profiles and hs-CRP were described in previous study [26].

#### PBMCs isolation and cell culture

PBMCs were isolated from the peripheral blood by ficoll density gradient centrifugation. PBMCs (1×10^6^ of the target cells per well) were cultured in complete RPMI 1640 containing 10% fetal bovine serum (FBS). The cells were demonstrated to have >95% viability with 2% Trypan blue exclusion. *trans*-4-[4-(3-Adamantan-1-ylureido)cyclohexyloxy] benzoic acid (*t*-AUCB) [27], a kind of sEHi, was synthesized in the laboratory of Dr. Bruce Hammock (UC Davis). At first, we prepared a 0.2 *M* stock solution by mixing 500μL of dimethylsulphoxide (DMSO) with 41.25 mg of t-AUCB. Then, stock solution was diluted with medium to different concentrations (0, 10, 50, 100 μmol/L) as required and used to treat cells for 24 h. While the PBMCs from the healthy subjects were cultured as the control without any intervention.

#### Real-time PCR

The cells were collected and total RNA was extracted from cells using TRIZOL kits as recommended by the manufacturer (Invitrogen). A total of 1 μg of total RNA isolated from each group using an RNeasy® kit (Qiagen) with the addition of DNase was reverse transcribed into cDNA and then 1 μl cDNA was used to perform real-time polymerase chain reaction assay (PCR). The primer sequences were as follows: 

FAS: F: 5^′^CGCGTGGCCGGCTACTCCTAC3^′^, R: 5^′^CGGCTGCCACACGCTCCTCT3^′^

IL-6: F: 5^′^CAATCTGGATTCAATGAGGAGAC3^′^, R: 5^′^CTCTGGCTTGTTCCTCACTACTC3^′^

GAPDH: F: 5^′^GGAAGGTGAAGGTCGGAGTCA3^′^, R: 5^′^GCTCCTGGAAGATGGTGATGG3^′^

PCR reactions were performed on the 7300 Real-Time PCR system using SYBR® GREEN PCR Master Mix (Applied Biosystems) as detailed in the manufacturer’s guidelines. Cycling parameters were 95°C for 10 sec, then 40 cycles of 95°C for 5 sec and 60°C for 31 sec. All the effective data were statistically analysed by the 2^-ΔΔCt^ method.

#### Western blotting

The cells were collected and total protein was extracted from cells using the kits as recommended by the manufacturer. Protein concentration was determined by the bicinchonininc acid (BCA) method, and samples were then loaded per well for sodium dodecyl sulfate polyacrylamide gel electrophoresis (SDS-PAGE). The proteins were electrophoretically transferred to polyvinylidene fluoride (PVDF) membranes. The membranes were blocked with blocking buffer, and incubated with primary antibodies, followed by incubation with secondary antibodies. At last, the bands were scanned by the GEL imaging system, and then the bands were analyzed using Photoshop software. All the bands were compared to β-actin as the internal control.

#### Statistical methods

All the data were analysed statistically using SPSS 16.0 software package. All results were expressed as the mean ± standard error (SE), except that hs-CRP results were logarithmically transformed to approximate a normal distribution. Single comparisons were examined with Student’s t-tests. One-way analysis of variance (ANOVA) was used to compare several groups. A linear relationship was assessed by least-square regression analysis. A two-sided *P* value of <0.05 was considered to be statistically significant.

## Results

### Basic clinical characteristics of the study subjects

There was no statistical significance between the ACS group and the control group in terms of gender, age, number of people who smoke, body mass index (BMI), blood pressure, fasting blood sugar (FBS), hemoglobin (Hb), serum creatinine(Cr), TG, TC, HDL-C, LDL-C (all *P* > 0.05). However, the total white blood cell count (WBC), troponin T (cTnT), creatine kinase (CK), creatine kinase isoenzyme (CK-Mb) and hs-CRP of the ACS group were higher than the control group (*P* < 0.05) (Table 1).

**Table 1:** **Basic clinical characteristics of the study subjects (**$\overset{―}{x}\pm \text{SE}$**)**

	**Control *(n = 65)***	**ACS *(n = 65)***	***P-Value***
Gender, male/female	39/26	38/27	0.542
Smoker, (n) (% of the total)	28 (43.1)	31 (47.7)	0.493
Age (years)	65.2 ± 10.2	64.8 ± 12.1	0.419
Systolic blood pressure (mmHg)	120.7 ± 14.2	123.4 ± 14.1	0.208
Diastolic blood pressure (mmHg)	74.6 ± 10.2	70.8 ± 7.4	0.285
TG (mmol/L)	1.8 ± 1.0	1.5 ± 1.0	0.390
TC (mmol/L)	4.4 ± 0.6	4.0 ± 0.8	0.154
HDL-C (mmol/L)	1.0 ± 0.3	0.9 ± 0.3	0.067
LDL-C (mmol/L)	2.6 ± 0.7	2.4 ± 0.8	0.265
Cr (μmol/L)	74.9 ± 17.0	82.7 ± 14.8	0.489
CK (U/L)	169.2 ± 21.1	207.3 ± 30.3^a^	0.038
CK-Mb (U/L)	13.5 ± 5.6	38.6 ± 43.0^a^	0.001
cTnT (ng/mL)	4.7 ± 4.5	795.7 ± 287.1^a^	0.000
FBS (mmol/L)	5.1 ± 0.9	5.3 ± 1.4	0.101
WBC (×10^9^/L)	6.9 ± 1.1	7.5 ± 2.4^a^	0.028
Hb (g/L)	123.0 ± 9.0	118.0 ± 13.0	0.347
BMI (Kg/m^2^)	22.8 ± 2.4	23.9 ± 3.0	0.086
h-CRP (mg/L)	1.3 ± 0.9	5.6 ± 4.1^a^	0.001
Medication history, n (%)			
ACEI/ARB	NE	15 (23.0)	-
β- blocker	NE	24 (36.9)	-
Statin	NE	30 (46.2)	-
CCB	NE	32 (49.2)	-
Insulin	NE	2 (3.0)	-

Hospitalized ACS patients were selected as the ACS group (n = 65) while the healthy normal subjects as the control group (n = 65). The levels of routine blood analyses, lipoproteins, fasting blood glucose, myocardial enzyme and high-sensitive C-reactive protein (hs-CRP) were determined within 24 hours after admission. Values are mean ± SE or percentage (n) (%). ^a^*P*<0.05, compared with control group.

### Expressions of FAS, IL-6 mRNA and protein in PBMCs and their relationships with hs-CRP

**Figure 1:**
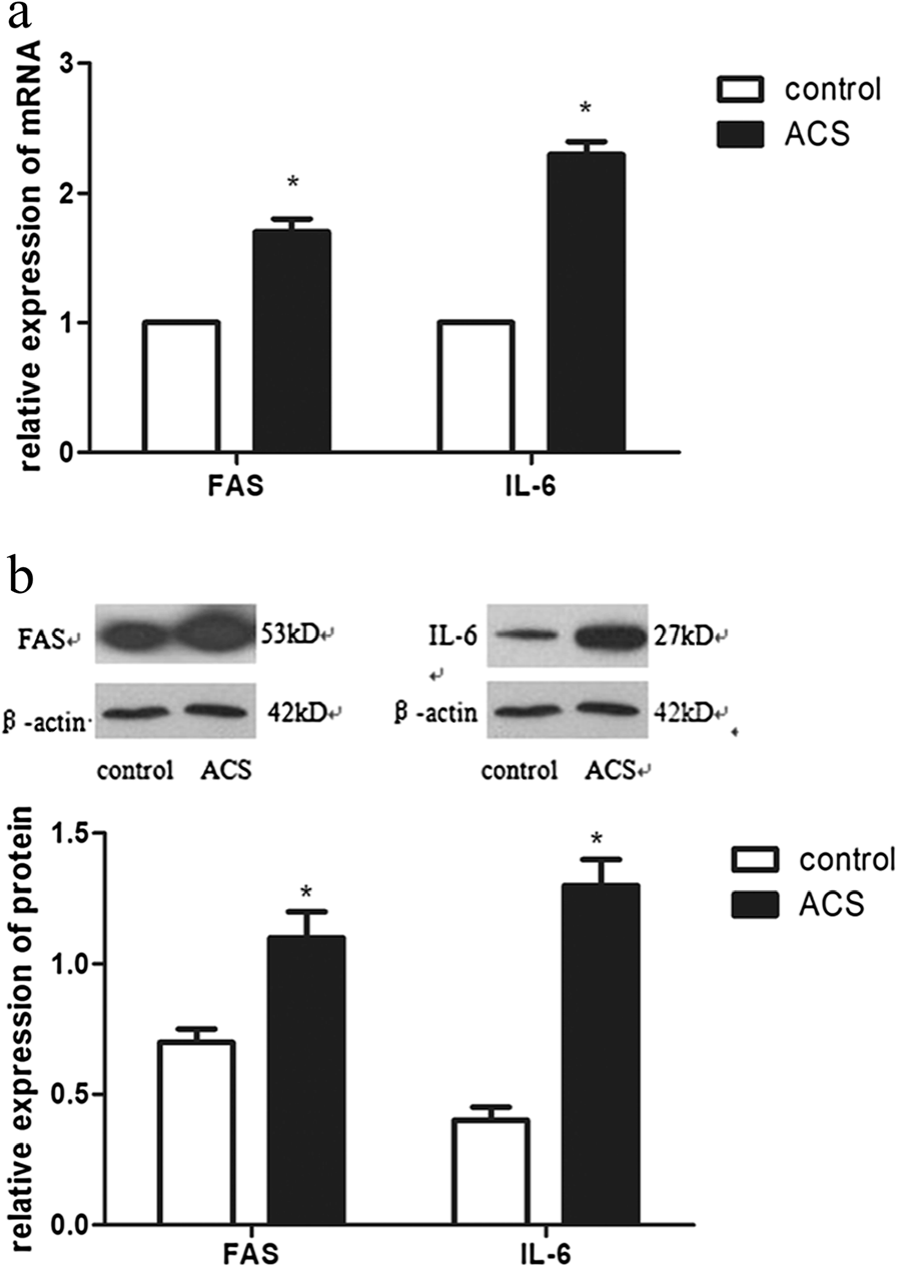
**Relative expression levels of FAS, IL-6 mRNA and protein in PBMCs.** PBMCs were isolated from peripheral blood of ACS patients. Healthy subjects were treated as control group. **a.** FAS and IL-6 mRNA were detected by realtime-PCR. All the effective data were statistically analyzed by the 2^-ΔΔCt^ method. **b.** FAS and IL-6 protein were detected by Western blotting. β-actin was the internal control. ^*^P<0.05 compared to the control group. FAS: fatty-acid synthase; PBMCs: peripheral blood mononuclear cells; ACS: acute coronary syndrome.

**Figure 2:**
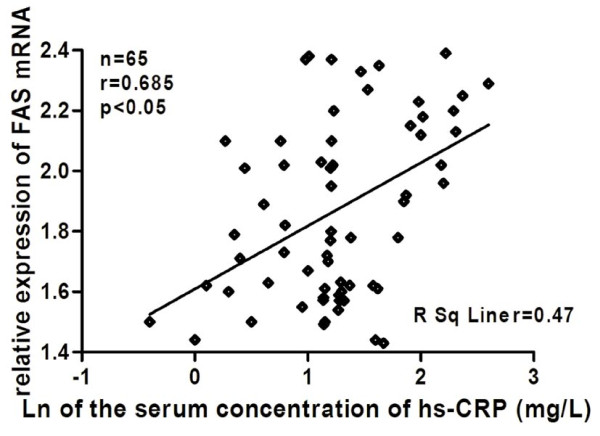
**Linear relationships between expression levels of FAS mRNA and the serum concentration of hs-CRP in ACS.** PBMCs were isolated from peripheral blood of ACS patients. FAS mRNA was detected by realtime-PCR. Hs-CRP was measured by immune nephelometry. Relative expression levels of FAS mRNA means its 2^-ΔΔCt^ value. Hs-CRP was log transformed to achieve normal distribution. The linear relationship between expression levels of FAS mRNA and serum concentration of hs-CRP was assessed by a least-square regression analysis. FAS: fatty-acid synthase; hs-CRP: high-sensitive C-reactive protein; ACS: acute coronary syndrome; PBMCs: peripheral blood mononuclear cells.

**Figure 3:**
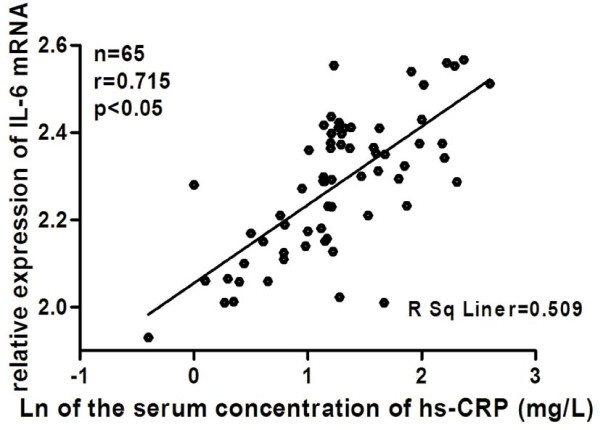
**Linear relationships between expression levels of IL-6 mRNA and the serum concentration of hs-CRP in ACS.** PBMCs were isolated from peripheral blood of ACS patients. IL-6 mRNA was detected by realtime-PCR. Hs-CRP was measured by immune nephelometry. Relative expression levels of IL-6 mRNA means its 2^-ΔΔCt^ value. Hs-CRP was log transformed to achieve normal distribution. The linear relationship between expression levels of IL-6 mRNA and the serum concentration of hs-CRP was assessed by a least-square regression analysis. FAS: fatty-acid synthase; hs-CRP: high-sensitive C-reactive protein; ACS: acute coronary syndrome; PBMCs: peripheral blood mononuclear cells.

As shown in Figure 1, compared with the control group, the mRNA and protein expression levels of FAS and IL-6 were significantly increased in the ACS group (all *P* < 0.05). Moreover, the levels of FAS and IL-6 mRNA were positively correlated with the serum concentration of hs-CRP in the ACS group (r = 0.685 *P* < 0.05 and r = 0.715 *P* < 0.05), respectively (Figure 2 and 3).

### Effects of sEHi on FAS and IL-6 mRNA in PBMCs

**Figure 4:**
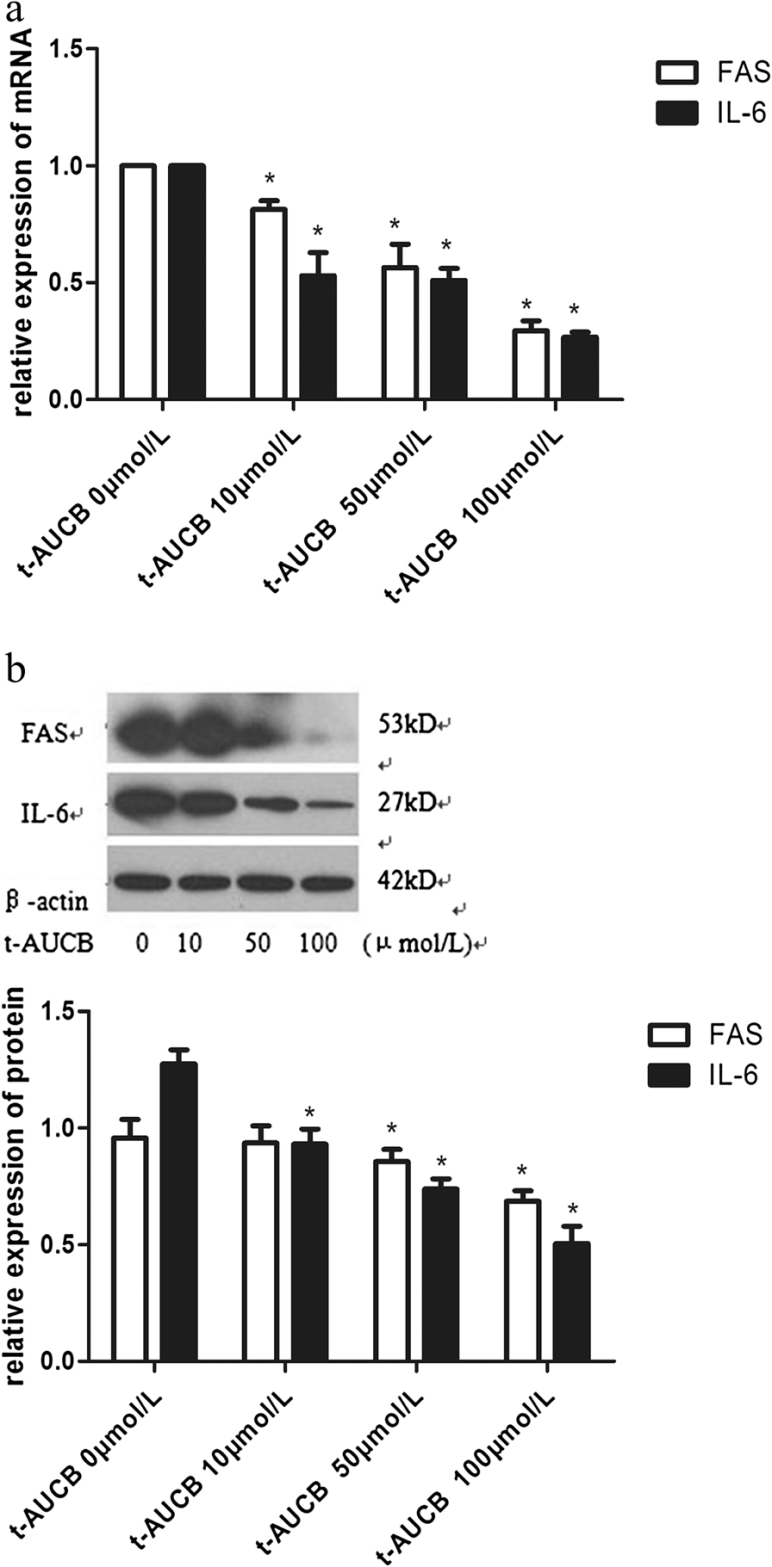
**Effects of sEHi on expression levels of FAS and IL-6 mRNA and protein in PBMCs.** PBMCs were isolated from peripheral blood of ACS patients. Then, *t*-AUCB, a kind of sEHi was added to cells of ACS group in various concentrations (0, 10, 50, 100) μmol/L and incubated for 24 hours. **a.** FAS and IL-6 mRNA were detected by real-time PCR. All the effective data were statistically analyzed by 2^-ΔΔCt^. **b.** FAS and IL-6 protein were detected by Western blots. β-actin was the internal control. ^*^P<0.05 compared to the group without sEHi (*t*-AUCB 0 μmol/L). sEHi: soluble epoxide hydrolase inhibitor; FAS: fatty-acid synthase; PBMCs: peripheral blood mononuclear cells; ACS: acute coronary syndrome.

*t*-AUCB had a dose-dependent inhibitory effect on the expression of FAS, IL-6 mRNA in PBMCs from the ACS group, and reached the maximum effect when the concentration of *t*-AUCB was 100 μmol/L. Compared with the control group (*t*-AUCB: 0 μmol/L), 10, 50, 100 μmol/L levels of *t*-AUCB had inhibited the expression of FAS, IL-6 mRNA in PBMCs from the ACS group with a statistical difference (*P* < 0.05) (Figure 4a).

### Effects of sEHi on FAS and IL-6 protein levels in PBMCs

*t*-AUCB had a dose-dependent inhibitory effect on the expression of FAS, IL-6 protein in PBMCs from the ACS group. Compared with the control group (*t*-AUCB: 0 μmol/L), levels of 10, 50, 100 μmol/L *t*-AUCB had inhibited the expression of FAS, IL-6 protein in PBMCs from the ACS group (*P* < 0.05). The relative expression levels of FAS protein in the *t*-AUCB 0, 10, 50 and 100 μmol/L groups were 0.957 ± 0.080, 0.935 ± 0.075, 0.855 ± 0.053, 0.685 ± 0.046, respectively. The relative expression of IL-6 protein in the *t*-AUCB 0, 10, 50 and 100 μmol/L groups were 1.276 ± 0.060, 0.9310 ± 0.064, 0.738 ± 0.044 and 0.506 ± 0.072, respectively (Figure 4b).

## Discussion

Numerous studies have demonstrated that the inflammatory level is increased in the ACS patients [11,12,28,29]. Shantsila and Lip [30] highlighted that monocytes were actively involved in the pathological processes related to ACS, which promoted the synthesis of pro-inflammatory molecules, such as IL-6, tumor necrosis factor-α (TNF-α) and hs-CRP. Among them, hs-CRP has been proved to be the strongest and most significant predictor of the inflammatory level and the risk of plaque instability and rupture [31-33]. Our studies showed that the serum concentration of hs-CRP and the total white blood cells were elevated in the ACS group, which was in accordance with previously published studies [34,35], indicated that inflammation was correlated with the development of cardiovascular events.

Among the inflammatory factors, IL-6 induces the production and secretion of CRP [36]. In the present study, we found that the expressions of IL-6 mRNA and protein in PBMCs were significantly increased in ACS group and then levels of IL-6 mRNA were positively correlated with the serum concentration of hs-CRP, which indicated that PBMCs were actived in the ACS group and more inflammatory factors were synthesized in cells.

The disruption of unstable coronary artery plaques is responsible for the majority of incidents of ACS [1-3,37]. FAS is a significant contributor to the rupture of atherosclerotic plaques. Firstly, increased SFA concentrations, which is inversely associated with cap thickness, might reflect a predisposition to rupture [7]. Results also showed that increased FAS in PBMCs promote synthesis of SFA [8]. Secondly, as already noted, the disrupted plaques are intimately related to the accumulation of lipid-filled macrophages at their edges. Macrophage cells produce cytokines that activate neighboring smooth muscle cells, resulting in extracellular matrix formation, fibrosis, and plaque instability, which play key roles in ACS [5,38,39]. FAS is also the key enzyme of the maturation of macrophages, as the uptake of modified lipoproteins is inhibited when fatty synthesis is suppressed during the differentiation process of the monocyte [9]. Therefore, FAS increase the occurence of ACS by regulating the synthesis of SFA and augmenting numbers of mature macrophages in the lipid core. Our results showed that, compared with the control group, the expression levels of FAS mRNA were significantly increased in the ACS group, which provided important evidence for the association between FAS and ACS.

A study showed that inflammation upregulated mRNA and protein expression of FAS, and stimulated lipogenesis in non-adipose tissues, causing ectopic lipid deposition [10]. We hypothesized that the composition of SFA in plaques was further increased as a result of upregulated FAS expression in the inflammatory state. Our studies proved that compared with the control group, the expression levels of FAS mRNA were positively correlated with the serum concentration of hs-CRP, which showed that the variation of fatty acid metabolism reflected high levels of inflammatory status *in vivo*. Therefore, it could be speculated that the expression of FAS in PBMCs was closely correlated with the vulnerable state of plaques and the inflammatory levels in the ACS patients.

Furthermore, our study also showed that the increased expression of FAS mRNA and protein in PBMCs from the ACS group were dose-dependently inhibited by sEHi. This result seems to be in agreement with a previous study in Mesenchymal stem cells (MSCs) which demonstrated that the decrease of FAS was dose dependent in MSCs treated with EETs [40]. In their study, they provided direct evidence that EETs induced increased expression of heme oxygenase-1 (HO-1) led to the increases in adiponectin, phosphorylation/inactivation of Acetyl-CoA carboxylase 1 (ACC1) and consequently decreased levels of FAS [40]. Most important, they concluded that increased expression of HO-1 might be a trigger for changes in lipid metabolism. HO-1, widely expressed in cells and tissues, is a rate-limiting enzyme that catabolizes heme and is important for the suppression of inflammatory responses [41]. Based on these data, we speculated the possible mechanism of our study was that sEHi lead to augmented circulation levels of EETs, which increased expression of HO-1, triggered a series reaction, consequently attenuated the levels of FAS expression. But the detail of the mechanism is unknown, and further studies are required.

Rae and Graham [14] showed that the C75, which was found to be an inhibitor of FAS [42], effectively blocked pro-atherogenic metabolic responses to a inflammatory factor, preventing this factor from inducing increases in macrophage triacylglycerol and cholesteryl ester content. It has been suggested that lipid accumulation induced by inflammation in cells could be reduced by inhibiting the synthesis of fatty acid by FAS. Moreover, the last study showed that induction of fatty acid synthesis by FAS was absolutely necessary for monocyte differentiation and the phagocytic activity of macrophages [9]. The inhibition of FAS could prevent lipoprotein uptake during monocyte differentiation [9], which was the crucial step of the maturation of macrophages. Additionally, it has been demonstrated previously that treatment with sEHi reduced the area of atherosclerotic lesions, and these effects were associated with a reduction of serum lipid and IL-6 [43].

IL-6 plays a significant role in the development of acute inflammatory responses, including endothelial and lymphocyte activation [44]. In our study, the increased expression of IL-6 mRNA and protein in PBMCs from the ACS group were inhibited by sEHi in a dose-dependent manner, which was consistent with the anti-inflammatory properties of sEHi in previous studies [22,43]. Resident macrophages would not produce pro-inflammatory proteins, such as TNF-α, IL-6, without nuclear factor kappa B (NF-κB) translocation to the nucleus [22]. Therefore, activated NF-κB was the underlying mechanism for elevated expression levels of IL-6 in PBMCs from patients with ACS. Furthermore, it was not difficult to deduce that anti-inflammatory properties of sEHi, especially lower expression levels of IL-6, might involve inhibition of NF-κB activation, though NF-κB activation was not measured directly in these studies. However, future studies need to elucidate the underlying mechanisms.

Some limitations of our study should be considered. Firstly, although SFA played an important role in the development of ACS, we did not monitor the SFA in plaques or plasma. In fact, we have realized the important role of measurement of SFA in plaques, however there are some difficulties: (1) as noted in our manuscript, we expected the concentration of SFA in plaques was reduced by regulating FAS, consequently decreased the occurrence of ACS. But it was impossible to get the plaques of ACS patients. (2) Afterwards, we figured out whether it was feasible to detect the concentration of SFA in plasma instead of plaques? But the answer is negative. Because the concentration of SFA in plasma was liable to be influenced by food metabolism. Moreover, a study showed that the concentration of SFA in plaques was not associated with it in plasma [7]. So it is not feasible to detect SFA in plasma instead of plaques. Taken together, we could not detect the concentration of SFA but speculated the reduction of SFA in plaques theoretically. Secondly, ACS encompass unstable angina, ST-elevation myocardial infarction (STEMI), and non-STEMI; however, we did not study the expression of FAS among these different categories of ACS. Thirdly, in our study, we studied the function of FAS *in vitro*, but the results *in vivo* remained unknown. Finally, the potential mechanisms underlying the observed effects were undefined.

## Conclusion

In summary, the present study showed that inhibition of sEH by *t*-AUCB reduced mRNA and protein expression of FAS and inflammatoty factor, IL-6, in PBMCs from the ACS group. These findings have led to the postulate that sEHi might attenuate the development of ACS by regulating lipid metabolism and inflammation as well as preventing rupture of atherosclerotic lesions.

## Competing interests

The authors declare that they have no competing interests.

## Authors’ contributions

All the authors were involved in the design of this study. XZ and JQD substantially contributed to the design of the study, performing the experiment, analysis of data, and drafting the manuscript. DYX and SPZ made contribution to design, analysis and revision of the manuscript. All the authors have read and approved the final version.
